# Detecting cancer recurrence in general practice: a Danish national cohort study

**DOI:** 10.3399/BJGP.2025.0174

**Published:** 2026-01-27

**Authors:** Kasper Grooss, Linda Aagaard Rasmussen, Kaj Sparle Christensen, Anette Fischer Pedersen, Larissa Nekhlyudov, Alina Zalounina Falborg, Peter Vedsted

**Affiliations:** 1 Research Unit for General Practice, Aarhus, Denmark; 2 Department of Public Health, Aarhus University, Aarhus, Denmark; 3 University Clinic for Innovative Patient Pathways, Department of Clinical Medicine, Aarhus, Denmark; 4 Brigham and Women’s Hospital, Harvard Medical School, Boston, MA, US; 5 Center for General Practice, Aalborg University, Aalborg, Denmark

**Keywords:** cancer survivors, cohort studies, general practice, neoplasm recurrence, referral and consultation, surveys and questionnaires

## Abstract

**Background:**

GPs may detect cancer recurrences between specialised visits or after completed follow-up. There is limited evidence on diagnostic routes to detecting cancer recurrence.

**Aim:**

To examine the level of GP involvement in cancer recurrence detection and to assess the time from the index consultation in general practice to a cancer recurrence diagnosis (diagnostic interval).

**Design and setting:**

This was a retrospective cohort study linking survey data from 667 Danish general practices with national register data.

**Method:**

Patients with a recurrence of seven cancer types diagnosed between 1 January 2022 and 31 May 2024 were included. The patients’ GPs provided details on how the recurrence was detected.

**Results:**

The GP survey achieved a response rate of 1265/2611 (48%). Of 1081 patients with recurrence, 478 (44%) first presented signs or symptoms of recurrence in general practice. Recurrence was diagnosed in 758 patients (70%) while they were under active specialised follow-up, and in 291 patients (27%) after completion of follow-up. Of 352 patients with a symptomatic presentation in general practice, the diagnostic pathway was initiated in general practice in 243 (69%), during subsequent follow-up in 57 (16%), and via other routes in 52 (15%). For patients with general practice-initiated diagnostic pathways, the 50th and 90th percentile of the diagnostic interval was 48 and 251 days for symptomatic presentations and 23 and 125 days for asymptomatic presentations, respectively.

**Conclusion:**

Four in 10 patients with cancer recurrence had their first point of contact in general practice. However, significant delays were observed. Improving detection pathways for cancer recurrence in general practice may benefit survivors across cancer types.

## How this fits in

Surveillance for cancer recurrence is typically managed in specialised clinics. General practice could play a greater role in cancer survivor care. Four in 10 patients with cancer recurrence first consulted their GP. GP actions depended on whether the patient was in active follow-up.

## Introduction

Advancements in primary cancer detection and treatment have improved survival rates, leading to a growing population of cancer survivors.^1^ Cancer survivor care is predominantly led by specialists, with a primary focus on surveillance for cancer recurrence.^2^ However, signs and symptoms of cancer recurrence often emerge between scheduled visits.^3–6^ GPs can detect and address early indications of cancer recurrence.^1^

Patients regularly consult their GP during cancer survivor care,^7^ and increased utilisation of general practice services has been observed for several months before a cancer recurrence diagnosis.^8^ This indicates that the GP already plays a role in cancer recurrence detection.

Although evidence linking early detection of cancer recurrence to improved survival is lacking for most cancer types, effective treatment options for recurrence and metastases are becoming available.^9,10^ Timely diagnosis of cancer recurrence may be increasingly important, as many local recurrences and select distant recurrences are treated with curative intent.^11^

The diagnostic interval, defined as the time from the first presentation of symptoms until diagnosis, is used to describe and measure the efficiency of patient pathways leading up to a cancer diagnosis.^12^

Existing research on cancer recurrence detection has focused on specific cancers using hospital records, but this approach is limited by small and selected cancer recurrence populations.^13^ However, GPs assess signs and symptoms in cancer survivors for all cancer types. To the authors’ knowledge, no studies have linked register data with GP-provided survey data to map the GPs’ role in cancer recurrence detection for multiple cancer types. A clear understanding of the current role of GPs in cancer recurrence detection may identify areas for improvement and assess if general practice could take a more formal role in cancer recurrence detection.

The aim of the study was to examine how often diagnostic pathways for cancer recurrence are started in general practice, and how often GPs initiate diagnostic investigation during and after specialised follow-up for cancer. An additional aim was to calculate the length of the diagnostic interval for pathways with an index consultation in general practice.

## Method

### Study design

A retrospective national cohort study using survey data from GPs linked with individual-level health register data was conducted.

### Setting

Denmark provides tax-funded health care with free access. Over 99% of residents are registered with a GP, who acts as gatekeeper to specialised health care. The GPs may refer patients to hospitals or clinics, transferring responsibility for diagnosis. In cancer patient pathways, investigations must begin within 14 days of referral. The GPs are required to keep detailed medical records of patient interactions, and they continuously receive and store secondary care discharge letters, test results, and outpatient clinic notes.^14^

### Participants

The authors of the current study sampled data on patients diagnosed with a first-time primary diagnosis of colorectal, lung, melanoma, breast, endometrial, ovarian, or bladder cancer (International Classification of Diseases [ICD]-10: C18–20, C34, C43, C50, C54, C56, C67) in 2012–2024 using the annually updated Danish Cancer Register^15^ and the Danish Colorectal Cancer Group Database^16^ for colorectal cancer. The continually updated Danish National Patient Register (DNPR)^17^ was used to sample the patients most recently diagnosed. Validated register-based algorithms were applied to identify patients with incident cancer recurrence between January 2022 and May 2024.^18–23^ A cancer recurrence was defined as the return of cancer at the original site or a distant location after curative treatment and a subsequent period with no register-based evidence of ongoing disease to indicate remission. This period was defined for each cancer based on clinical expert opinion: 90–180 days after surgery or 30–90 days after the last day of oncological treatment, whichever came last.^18–23^ The cancer recurrence indicators included diagnosis and procedure codes from the DNPR and pathology test results from the Danish National Pathology Register.^24^

### Data collection

Questionnaire data were collected from January 2023 until July 2024. The registered general practice of each patient case received a questionnaire invitation. The Danish National Health Service Register^25^ provided information on registered general practice. Initially, GPs of patients with cases diagnosed within 12 months were invited. Subsequently, invitations were sent every 4 months to GPs of newly identified patient cases. The questionnaire was completed with support from the patients’ medical records. Non-responders received a reminder after 3 weeks. Responders were remunerated with DKK 146 (EUR 20).

### Variables and data sources

From the GP questionnaire, information on three key variables was obtained:

- symptom presentation in general practice: ‘Based on your current knowledge, did the patient present symptoms or signs of illness in general practice in the months leading up to the recurrence diagnosis?’ (yes/no/don’t know);
- the place of diagnostic initiation: ‘Where was the recurrence diagnosis initiated?’ (general practice/specialised follow-up/patient contacted hospital/patient contacted private specialist/out-of-hours services/during hospitalisation/other way/don’t know); and
- follow-up status: ‘Was the patient enrolled in active specialised follow-up when the recurrence was diagnosed?’ (yes/no/don’t know).

Patients were categorised as having an index consultation in general practice if the GP ticked ‘yes’ under ‘symptom presentation in general practice’ or ticked ‘general practice’ under ‘place of diagnostic initiation’. These patients were then assigned to one of three diagnostic pathways based on the GPs’ responses:

- symptomatic and diagnostics by GP;
- symptomatic and diagnostics by non-GP; and
- asymptomatic and diagnostics by GP.

The GP provided the index consultation date: ‘When did you/your practice first see the patient with symptoms or signs that — based on your current knowledge — could have been attributed to cancer recurrence?’

Diagnostic intervals were calculated as the time between index consultation and cancer recurrence diagnosis. Interval length was truncated at 365 days (*n* = 11). Negative values of up to –100 days were adjusted to 0 days (*n* = 27), as they were likely because of inaccurate cancer recurrence diagnosis dates. If a negative interval length was longer than –100 days (*n* = 38) or the interval length exceeded 365 days and the GP reported ‘no delay’ (*n* = 15), the interval was coded as ‘missing’ as it was likely caused by an erroneous GP-specified date.

Comorbidity was defined according to the Charlson Comorbidity Index (CCI)^26^ based on DNPR diagnosis codes from hospital contacts in the 10 years preceding the cancer recurrence diagnosis, excluding cancer-related diagnoses. CCI scores were categorised into ‘low’ (score 0), ‘medium’ (scores 1–2), and ‘high’ (scores >2).

Statistics Denmark^27^ provided information on educational level and cohabitation status. Educational level was categorised based on years of education according to the International Standard Classification of Education^28^ into ‘low’ (<10 years), ‘medium’ (10–15 years), and ‘high’ (>15 years). Cohabitation status was categorised as ‘cohabitating’ (married or registered with a partner) or ‘living alone’ (widowed, divorced, or unmarried).

### Statistical methods

The place of diagnostic initiation was described by the total number and percentage within categories. Generalised linear models for the binomial family were used to estimate crude and adjusted prevalence ratios. To identify confounders for inclusion in the adjusted analyses, a directed acyclic graph was used (Supplementary Figure S1).^29^ Analyses were restricted to patients with complete data on all variables required for that analysis.

Quantile regression^30^ was used to estimate crude and adjusted diagnostic intervals in calendar days at the 50th and 90th percentiles. Diagnostic intervals were derived by counting days using the ‘qcount’ procedure.^31,32^ Diagnostic interval differences were calculated as marginal effects. All analyses were performed with Stata statistical software, version 18.0.

## Results

### Participants

**Figure 1.**
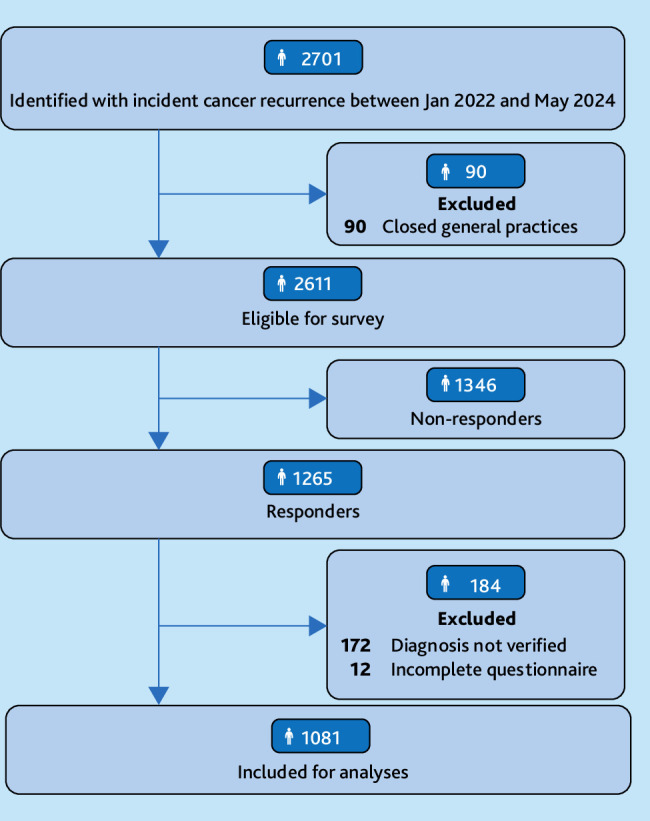
Study population flowchart.

The algorithms identified 2701 patients. In total, 1081 patients with cancer recurrence (40%) from 667 general practices (Figure 1) were included.

Patient characteristics were similar for patients registered with responding and non-responding GPs. Table 1 shows the characteristics of the study population. One-third was aged ≥75 years, two-thirds were female patients, breast cancer was the most frequent cancer type (392/1081, 36%), and 756/1081 (70%) had a low comorbidity level.

The median time from primary cancer diagnosis to cancer recurrence was 2.9 years (interquartile interval [(IQR] 1.2–5.9), and the median diagnostic interval was 49 days (IQR 20–121).

**Table 1.** Characteristics of study population with incident cancer recurrence (January 2022 to May 2024) (*N* = 1081) and diagnosis verified by the GP

**Characteristic**	**Study population, *n* (%)**
Total	1081 (100)
Cancer type	
Breast	392 (36)
Colorectal	265 (25)
Lung	172 (16)
Melanoma	133 (12)
Bladder	51 (5)
Endometrial	39 (4)
Ovarian	29 (3)
Sex	
Male	366 (34)
Female	715 (66)
Age group (years)	
18–54	161 (15)
55–64	211 (20)
65–74	338 (31)
≥75	371 (34)
Educational level^a^	
Low	335 (31)
Medium	489 (45)
High	257 (24)
Civil status	
Cohabitating	612 (57)
Living alone	469 (43)
Comorbidity level^b^	
Low	756 (70)
Medium	276 (26)
High	49 (5)

^a^According to the International Standard Classification of Education divided into low (<10 years), medium (10–15 years), and high (>15 years).^b^According to the Charlson Comorbidity Index score, calculated based on diagnosis codes registered in the Danish National Patient Register in the 10 years preceding a recurrence diagnosis (excluding cancer-related diagnoses), divided into low (score 0), medium (score 1–2), and high (score ≥3).

### Diagnostic pathways

Of 1081 patients, 44% (*n* = 478) had their index consultation in general practice: 352 symptomatic presentations, 118 asymptomatic presentations, and eight unknown symptom presentations. The diagnostic pathway was initiated by the GP in 369 patients.

**Figure 2.**
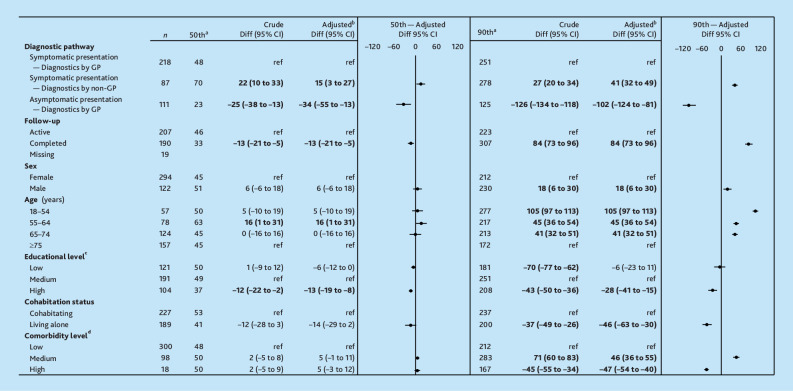
Differences in length of diagnostic interval in calendar days at the 50th and 90th percentiles for patients with an index consultation in general practice.Patients with missing diagnostic interval were omitted (*n* = 62) (*N* = 416). Bold indicates statistical significance, *P*≤0.05. ^a^Pseudo-centiles used for crude diagnostic interval length. ^b^Diagnostic pathway was adjusted for follow-up, sex, age, educational level, cohabitation status, and comorbidity level. Comorbidity level was adjusted for educational level and age. Educational level and cohabitation status were adjusted for age. Follow-up, sex, and age were not adjusted. ^c^According to the International Standard Classification of Education, divided into low (<10 years), medium (10–15 years), and high (>15 years). ^d^According to the Charlson Comorbidity Index score, based on diagnosis codes in the Danish National Patient Register in the 10 years preceding a recurrence (excluding cancer-related diagnoses), divided into low (score 0), medium (score 1–2), and high (score ≥3). CI = confidence interval. diff = difference.

The diagnostic interval varied in length by patient characteristics at the 90th percentile (Figure 2). The largest difference was observed in patients aged 18–54 years with 105 days longer intervals (95% confidence interval [CI] = 97 to 113) compared with patients ≥75 years of age.

### Symptomatic presentations

**Figure 3.**
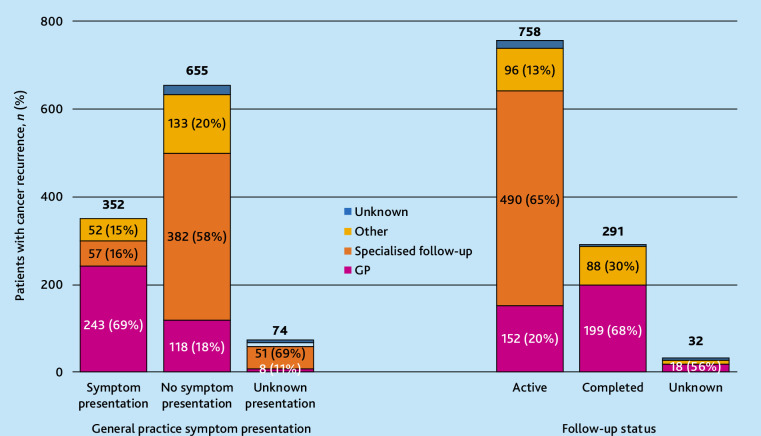
Place of diagnostic initiation categorised by symptom presentation in general practice and follow-up status (*N* = 1081). ‘Other’ includes five categories: contacted hospital, consulted private specialist, consulted out-of-hours services, diagnostics initiated during a hospital stay, and other diagnostic route.

Out of the 352 patients presenting symptoms in general practice, the diagnostic pathway was initiated in general practice for 243 (69%), in specialist follow-up for 57 (16%), and in other routes for 52 (15%) (Figure 3). When the GP initiated the diagnostic pathway, the diagnostic interval was 48 days at the 50th percentile and 251 days at the 90th percentile.

When the GP did not initiate the diagnostic pathway, the interval was 15 days longer (95% CI = 3 to 27) at the 50th percentile and 41 days longer (95% CI = 32 to 49) at the 90th percentile compared with when the GP did initiate the diagnostic pathway (Figure 2).

### Asymptomatic presentation

Patients aged 18–54 years were 1.6 times more likely (95% CI = 1.0 to 2.2) to be detected through an asymptomatic presentation in general practice compared with patients ≥75 years (Table 2). No evidence suggests that other covariates affected the likelihood of an asymptomatic presentation.

**Table 2.** Prevalence ratios of asymptomatic presentation compared with symptomatic presentation in general practice for patients with diagnostics initiated by the GP (*N* = 369)

**Characteristic**	**Patient,** ***n***	**Adjusted^a,b^ prevalence ratio (95% CI)**
**Follow-up status**		
Active	152	Reference
Completed	199	1.2 (0.9 to 1.7)
Missing	18	
**Sex**		
Female	258	Reference
Male	111	0.8 (0.5 to 1.1)
**Age** (years)		
18–54	49	**1.6 (1.0 to 2.2)**
55–64	73	1.1 (0.7 to 1.6)
65–74	111	1.2 (0.8 to 1.7)
≥75	136	Reference
**Educational level^c^**		
Low	107	0.9 (0.6 to 1.3)
Medium	170	Reference
High	92	1.3 (0.9 to 1.7)
**Cohabitation status**		
Cohabitating	204	Reference
Living alone	165	0.9 (0.7 to 1.2)
**Comorbidity level^d^**		
Low	274	Reference
Medium	81	0.8 (0.5 to 1.1)
High	14	0.4 (0.1 to 1.6)

^a^Crude estimates were similar to adjusted estimates and, therefore, omitted from the table.^b^Comorbidity level was adjusted for educational level and age. Educational level and cohabitation status were adjusted for age. Follow-up, age, and sex were not adjusted.^c^According to the International Standard Classification of Education, divided into low (<10 years), medium (10–15 years), and high (>15 years).^d^According to the Charlson Comorbidity Index score, calculated based on diagnosis codes registered in the Danish National Patient Register in the 10 years preceding a recurrence diagnosis (excluding cancer-related diagnoses), divided into low (score = 0), medium (score = 1–2), and high (score ≥ 3). Bold indicates statistical significance, *P*≤0.05. CI = confidence interval.

When the GP initiated the diagnostic pathway, the diagnostic interval was 34 days shorter (95% CI = 13 to 55) at the 50th percentile and 102 days shorter (95% CI = 81 to 124) at the 90th percentile compared with symptomatic presentations (Figure 2).

### Follow-up

Cancer recurrence was detected during active follow-up in 758 of 1081 (70%) patients and after completed follow-up in 291 (27%) patients (Figure 3). The GP initiated the diagnostic pathway for 20% (152/758) of patients detected during active follow-up and for 68% (199/291) of patients detected after completed follow-up.

For patients with a symptomatic presentation in general practice, GPs were 1.6 times more likely (95% CI = 1.4 to 1.9) to initiate the diagnostic pathway after completed follow-up compared with during active follow-up (Table 3). No other covariates affected the likelihood of GPs initiating the diagnostic pathway for symptomatic presentations.

Patients where recurrence was detected after completed follow-up had 13 days shorter intervals (95% CI = 5 to 21) at the 50th percentile and 84 days longer intervals (95% CI = 73 to 96) at the 90th percentile compared with patients detected during active follow-up (Figure 2).

**Table 3.** Prevalence ratios of diagnostics initiated by the GP compared with diagnostics initiated by non-GP for patients with a symptomatic presentation of cancer recurrence in general practice (*N* = 352)

**Characteristic**	**Patient,** ***n***	**Adjusted^a,b^** ^**,**^ **prevalence ratio (95% CI)**
**Follow-up status**		
Active	194	Reference
Completed	141	**1.6 (1.4 to 1.9)**
Missing	17	
**Sex**		
Female	236	Reference
Male	116	1.0 (0.7 to 1.3)
**Age** (years)		
18–54	45	0.8 (0.5 to 1.3)
55–64	71	1.0 (0.7 to 1.4)
65–74	101	1.0 (0.7 to 1.3)
≥75	135	Reference
**Educational level^c^**		
Low	109	0.9 (0.8 to 1.2)
Medium	161	Reference
High	82	0.9 (0.7 to 1.2)
**Cohabitation status**		
Cohabitating	192	Reference
Living alone	160	1.0 (0.9 to 1.2)
**Comorbidity level^d^**		
Low	245	Reference
Medium	88	0.9 (0.8 to 1.1)
High	19	0.9 (0.6 to 1.2)

^a^Crude estimates were similar to adjusted estimates and, therefore, omitted from the table.^b^Educational level and cohabitation status were adjusted for age. Comorbidity was adjusted for educational level and age. Follow-up, age, and sex were not adjusted.^c^According to the International Standard Classification of Education, divided into low (<10 years), medium (10–15 years), and high (>15 years).^d^According to the Charlson Comorbidity Index score, calculated based on diagnosis codes registered in the Danish National Patient Register in the 10 years preceding a recurrence diagnosis (excluding cancer-related diagnoses), divided into low (score = 0), medium (score = 1–2), and high (score ≥ 3). Bold indicates statistical significance, *P*≤0.05. CI = confidence interval.

## Discussion

### Summary

Four in 10 patients had an index consultation in general practice, and one-third of diagnostic pathways were initiated by the GP. Seven in 10 patients were detected during active follow-up. The GP was less likely to initiate diagnostic investigations in patients presenting symptoms during active follow-up than after completed follow-up. The shortest diagnostic intervals in this study were observed when the GP initiated diagnostic investigations for asymptomatic presentations. The longest diagnostic intervals were seen in patients aged 18–54 years, for cancer recurrence detected after completed follow-up, and in patients with low comorbidity.

### Strengths and limitations

Identifying patients with cancer recurrence is a key challenge in cancer recurrence research, as recurrences are not routinely registered. Register-based algorithms enabled us to leverage the Danish health registers to identify a national cohort of patients with recurrence across seven cancer types with high accuracy.

Collecting survey data from the GPs offered significant advantages. The study mitigated the potential effects of patient health and socioeconomic deprivation on study participation while enabling patient inclusion even in the event of patient death. Recall bias was limited and data quality was improved by leveraging the GPs’ access to detailed medical records, including hospital discharge letters and outpatient clinic notes.

The study design was susceptible to selection and information bias. The algorithms may have missed patients with cancer recurrence and falsely included patients without recurrence. The GPs verified cancer recurrence diagnoses, which minimised the risk of false positives. Algorithm sensitivities ranged from 83% to 97%, indicating some missed patient cases, while specificities ranged from 90% to 99%. Patients with comorbidities and those who were frail may be at higher risk of being missed by the algorithms as their health state may contraindicate indicators of cancer recurrence, such as biopsy or cancer treatment.^18–23^ However, including patients without an incentive to expedite diagnosis could reduce the clinical relevance of the study’s findings.

Although the GP response rate of 1265/2611 (48%) was notably higher than recent comparable surveys in Danish general practices,^33,34^ it was lower than desired and led to reduced statistical precision. Although the patient characteristics were similar between responding and non-responding GPs across covariates, non-responder bias cannot be ruled out. Some GPs might have chosen not to respond if they had contributed to undue delays, which could have introduced selection bias and underestimation of the diagnostic interval. As the GPs were anonymous and adverse events and trajectories could also motivate GP participation, such selection bias is unlikely to have significantly affected the results. Additionally, GPs might have responded in their favour and misclassified early symptoms as unrelated to the cancer recurrence, which could have led to underestimated numbers of symptomatic presentations in general practice.

### Comparison with existing literature

Previous studies found that cancer recurrence was detected outside of follow-up in 44–47% of cases of patients with breast cancer and 42% of cases of patients with colorectal cancer.^3–6^ These findings align with the current study, indicating that GPs play a significant role in cancer recurrence detection.

The literature on diagnostic intervals is sparse, and direct comparisons between studies and countries are difficult.^12^ To the authors’ knowledge, no study has investigated diagnostic intervals for cancer recurrence in patients detected outside of follow-up. A Danish study reported diagnostic intervals for patient pathways for primary colorectal cancer initiated in general practice.^35^ They found a median diagnostic interval of 40 days, which is comparable with the 48 days in the current study for symptomatic presentations. Longer intervals may be because of mistaking cancer recurrence symptoms for late effects of cancer treatment.^6^

In the current study, patients living alone were less likely to experience prolonged diagnostic intervals. Previous studies have indicated that patients living alone are less likely to seek timely medical care when experiencing alarm symptoms for a primary cancer^36^ but they are less likely to experience the most extended patient and doctor delays.^36,37^

A dose–response relationship was observed in the current study between age and length of diagnostic interval at the 90th percentile, with younger patients experiencing longer intervals. This finding is similar to findings for primary cancer.^38,39^ It may reflect greater symptom resilience in younger patients, making them less inclined to seek GP re-evaluation, as well as fewer asymptomatic presentations because of less frequent testing in general practice.^40^

### Implications for practice

The role of primary care has often been perceived as marginal in cancer recurrence detection.^1^ The findings reveal that the diagnostic pathways frequently begin in general practice during and after completed follow-up.

The GP did not initiate diagnostic investigations for a third of patients with symptomatic presentation in general practice. These missed opportunities suggest room for improvement and call for a systematic approach to cancer recurrence detection in general practice.^41^

The GPs were less likely to initiate diagnostic investigations of patients with symptomatic presentation in active follow-up. Specialised follow-up visits may serve as a safety net that patients and GPs are attentive to.^42^ This would explain why patients in active follow-up had longer diagnostic intervals at the 50th percentile and shorter intervals at the 90th percentile. The findings indicate that GPs need to recognise their role in timely diagnosis of recurrence during active follow-up.^43^

The shortest diagnostic intervals were observed when GPs initiated diagnostics for asymptomatic presentations. This is likely because of incidental findings prompting immediate diagnostic investigations rather than a stepwise approach for symptomatic presentations.^44^ This may also explain the shorter diagnostic intervals for patients with high comorbidity levels, as they have more general practice contacts with routine tests compared with patients with low comorbidity levels.^45^ The asymptomatic presentation group comprised a third of all GP-initiated pathways. This highlights the frequent interaction between GPs and cancer survivors, and the importance of health monitoring for this population.^8^

These findings suggest that GPs play an essential role in detecting and addressing potential signs of cancer recurrence, underscoring the importance of active and continuous involvement of GPs in cancer survivor care. The large proportion of index consultations in general practice advocates the need for a systematic approach to guide GPs on when and how to initiate diagnostics for cancer survivors.

Evidence is needed to support the development of a systematic approach to cancer recurrence detection in general practice.

## Data

The technical appendix and statistical codes are available from the corresponding author. Owing to Danish data protection legislation, the dataset is accessible only to pre-approved collaborative partners.
